# Understanding Dengue Underreporting: An Analysis of the Impacts for the World, Latin America and Brazil

**DOI:** 10.1155/tswj/9993911

**Published:** 2025-07-16

**Authors:** Carlos Letacio Silveira Lessa, Bianca Sampaio Dotto Fiuza, Katharine Valéria Saraiva Hodel, Cíntia Minafra, Marilda de Souza Gonçalves, Bruna Aparecida Souza Machado

**Affiliations:** ^1^Postgraduate Program in Industrial Management and Technology, SENAI CIMATEC University Center, Salvador, Brazil; ^2^Gonçalo Moniz Institute, Oswaldo Cruz Foundation (IGM-FIOCRUZ/BA), Salvador, Brazil; ^3^SENAI Institute of Innovation (ISI) in Health Advanced Systems (CIMATEC ISI SAS), University Center SENAI/CIMATEC, Salvador, Brazil; ^4^School of Veterinary and Animal Science, Federal University of Goiás, Goiânia, Brazil

**Keywords:** dengue, surveillance, underreporting

## Abstract

Notifiable diseases are legally designated due to their epidemic potential, requiring mandatory reporting by healthcare professionals to support public health surveillance and control. This narrative review analyzes weaknesses in the compulsory notification process, with emphasis on dengue, revealing critical gaps in surveillance systems and health data reporting globally. A structured literature search was conducted in PubMed, Scopus, and SciELO, including articles in English, Portuguese, and Spanish. Inclusion criteria focused on studies related to dengue surveillance, underreporting, health information systems, and notification policies. The findings demonstrate persistent failures in data collection, diagnostic confirmation, and reporting practices, which compromise the accuracy of epidemiological indicators and hinder timely responses. These limitations reflect broader challenges in dengue control. Strengthening surveillance infrastructure and reporting protocols is essential to mitigate underreporting and improve decision-making in public health.

## 1. Introduction

Arboviruses have been affecting the human population for hundreds of years [1]. Dengue fever is one of the arboviral diseases with the greatest impact on different health systems, especially in tropical locations. The dengue virus (DENV), which is transmitted mainly by the female mosquitoes *Aedes aegypti* and *Aedes albopictus*, causes this [2]. Three subspecies of *Aedes aegypti* have already been identified: *Aedes aegypti aegypti*, which is widely distributed in tropical and subtropical regions due to human activity*; Aedes aegypti formosus*, found in sub-Saharan Africa; and *Aedes aegypti queenslandensis*, found in the Mediterranean, Australia, and parts of East Africa [3, 4]. The occurrence of these subspecies of *Aedes aegypti* has had an impact on different public health actions, resulting in a reduction in the effectiveness of vector control strategies [5]. The advancement of civilization, characterized by deforestation and urbanization leading to ecosystem disruption, has significantly altered the natural habitats of mosquitoes, the primary vectors of this disease [6, 7]. Consequently, the interaction between humans and disease vectors has become increasingly detrimental to human health [8].

The earliest state interventions in disease prevention and control, based on modern scientific principles, trace back to the early 20th century [9, 10]. These efforts were influenced by the advancements of the bacteriological era and the understanding of the epidemiological cycles of various infectious and parasitic diseases [11]. Such interventions primarily involved orchestrating extensive sanitation campaigns aimed at controlling diseases that posed significant threats to economic activities, including yellow fever, plague, and smallpox [12]. The campaigns employed precise instruments for case diagnosis, vector control, immunization, and mass drug treatment, among others [13, 14]. The operational model was based on vertical actions, strongly inspired by the military, and comprised well-established phases—preparatory, attack, consolidation, and maintenance [15].

The surveillance system plays a crucial role in the prevention and control of infectious diseases with high contamination and morbidity/mortality characteristics for the population [16, 17]. Since dengue is a significant disease, it must be monitored and controlled due to its infectious and emergent nature [18]. Thus, as indicated in the literature, notification has highlighted the need for improvement in data collection processes for surveillance [19].

Notification is a legal mechanism aimed primarily at identifying, controlling, and planning actions against diseases and health problems [20]. The use of this data will be important for conducting efficient diagnosis of events that occur in each population, providing elements to support information for knowledge, planning, and decision-making regarding diseases and health problems [21].

Underreporting has been the greatest ally in the spread of arboviruses, specifically dengue [22]. The absence of symptoms or mild symptomatology and similarities with other arboviruses have made it difficult for healthcare professionals to diagnose the specific disease based on symptoms [23]. Additionally, difficulties in completing notification forms by healthcare professionals have caused serious problems in disease control strategies [24]. The statistics, crucial for implementing contingency and combat actions against diseases, are significantly affected by underreporting, especially of milder and asymptomatic cases [25]. This increases the socioeconomic implications that the disease may bring to these populations and restricts preventive control interventions over time [24].

The viruses transmitted by the vector have caused a significant impact on human population health [26]. Dengue has attracted attention and concern due to its endemic and epidemic presentation in tropical and subtropical regions with extremely aggressive and alarming patterns, affecting approximately 390 million people annually, with approximately 96 million resulting in clinical manifestations [18, 27, 28]. For example, in Mexico, dengue has been responsible for approximately 139,000 symptomatic infections, which are 4.5 times higher than officially reported cases, demonstrating a significant number of underreported cases [29]. Similarly, in Southeast Asia, while the average annual dengue cases are 386,000, it is believed that this average could reach almost 3 million episodes [30]. The underreporting of dengue cases has also been reported in different countries on the African continent [31]. This brings serious losses to health action planning, determining human, social, and economic losses with projections of costs around 170 million per year, including surveillance and vector control costs [32].

This study is aimed at reaffirming the importance of compulsory notification for public health planning and actions to control infectious diseases characterized by the severity and dissemination potential of the causative agent. Thus, aspects related to dengue are addressed, focusing on epidemiology (with a focus on Latin America and Brazil), strategies for control, factors related to monitoring cases, and the impacts of underreporting.

## 2. Materials and Methods

This study consists of a narrative literature review, designed to explore the impacts of dengue underreporting and the implications for public health surveillance, particularly in Brazil and Latin America. The objective was to consolidate relevant knowledge on the epidemiology of dengue, factors contributing to notification failures, and the resulting challenges for disease control and health planning. The literature was selected based on a nonsystematic search strategy, carried out between January and April 2024. The search included peer-reviewed articles, governmental and intergovernmental reports, and official epidemiological bulletins, primarily from databases such as PubMed, Scopus, SciELO, as well as documents from the World Health Organization (WHO), Pan American Health Organization (PAHO), European Centre for Disease Prevention and Control (ECDC), and the Brazilian Ministry of Health.

Search terms included combinations of “dengue”, “underreporting”, “notification”, “surveillance”, “epidemiology”, “public health”, and “Latin America” in English, Portuguese, and Spanish. The inclusion criteria considered studies published from 2000 to 2024 that addressed at least one of the following: dengue surveillance systems, data quality and completeness, epidemiological trends, economic and health impacts of underreporting, and legal or political frameworks for compulsory notification.

The review integrated scientific articles, technical publications from health agencies, and narrative or systematic reviews, highlighting both global perspectives and national experiences, especially from Brazil. No formal protocol (e.g., PRISMA) was followed, as the aim was not to perform an exhaustive or quantitative synthesis but to provide a broad and critical overview of the topic. The analysis was conducted through thematic synthesis, grouping the findings into four main areas: (1) dengue epidemiology and transmission dynamics, (2) surveillance system structures and performance, (3) factors associated with underreporting, and (4) health and policy implications.

Limitations of this review include the narrative nature of the synthesis, the potential for selection bias, and the lack of a formal quality appraisal of individual sources. Nonetheless, this approach allowed a comprehensive integration of different types of evidence and supported the identification of key gaps and challenges in dengue notification and control systems.

## 3. Dengue Epidemiology

Dengue, a mosquito-borne viral disease, has emerged as a critical global health concern [33]. Historically, it has been a significant burden in tropical and subtropical regions, with over half of the world's population at risk of infection [34]. The disease is caused by four distinct serotypes of the virus, classified under the Flaviviridae family and *Flavivirus* genus, known as DENV-1, DENV-2, DENV-3, and DENV-4 [35]. These serotypes are not only responsible for widespread epidemics but are also linked to the more severe manifestations of the disease [36].

Clinically, dengue presents a broad spectrum of symptoms, ranging from mild fever to severe complications such as dengue hemorrhagic fever (DHF) and dengue shock syndrome (DSS) [37]. However, the heterogeneous nature of dengue often leads to misdiagnosis or underreporting, particularly in regions endemic to other febrile illnesses [38]. Dengue, a viral disease with a storied past, has emerged as a significant global health concern, affecting millions annually across diverse regions [39].  Table 1 summarizes the epidemiological findings in Brazil, Latin America, and globally.

## 4. Control Strategies to Counter DENV

**Figure 1:**
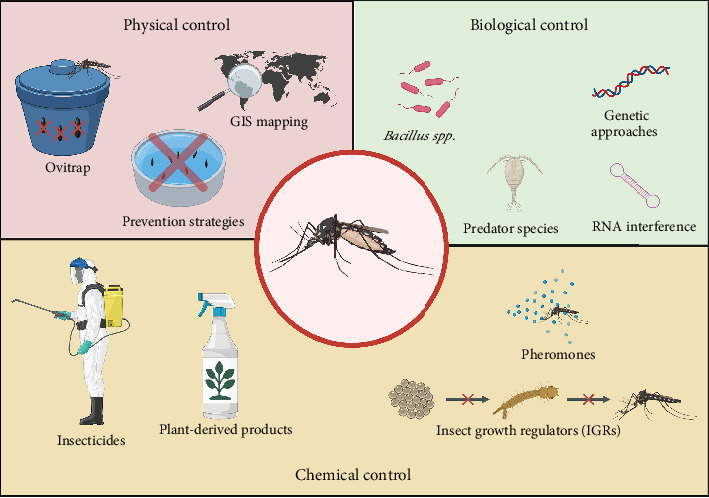
Integrated vector control strategies for *Aedes* mosquitoes, categorized by physical, biological, and chemical approaches. Methods include environmental management (e.g., ovitraps and GIS mapping), biological control (e.g., *Bacillus thuringiensis* and RNA interference), and chemical tools (e.g., insecticides and insect growth regulators). Image created with Biorender.com (accessed on May 15, 2025).

Dengue poses a significant global health threat, necessitating ongoing efforts to develop and implement effective control measures. The multifaceted nature of dengue transmission demands integrated control strategies that address both larval and adult mosquito populations. By combining physical, biological, and chemical control methods, stakeholders can enhance the efficacy and sustainability of dengue control programs.  Figure 1 shows an overview of the main strategies for controlling the vector and DENV transmission.

### 4.1. Physical Control Strategies

Recent studies have underscored the importance of understanding the behavioral patterns of *Aedes aegypti* mosquitoes for effective control. Wong et al. [54] demonstrated the efficacy of targeting oviposition sites to reduce mosquito populations, with advancements such as lethal ovitraps showing promise in capturing gravid females [55, 56]. Innovative designs, such as the Gravid Aedes Trap (GAT) and Aedes Gravid Ovitrap (AGO), offer larger volumes and incorporate attractants to enhance trapping efficiency [57]. Geographical information system (GIS) provides a powerful means to analyze surveillance data by visualizing disease patterns and their relationship with the environment [58]. In the health sector, GIS correlates diverse datasets with different environments, aiding survey planning and identifying breeding sites for disease vectors [59]. Dengue surveillance benefits from GIS by enabling the visualization of disease distribution and trends over time, facilitating targeted resource allocation for disease control measures [58]. Environmental control is also a tool linked to physical control. It is based on environmental modification or manipulation, as well as changes in habitat or human behavior [55]. Different initiatives are associated with environmental control, including improving the water supply and system, solid waste management, the routine and quality of cleaning carried out by the community, and modifications to building structures to prevent the accumulation of standing water [56].

### 4.2. Chemical Control Strategies

While insecticides remain a cornerstone of mosquito control, efforts are underway to mitigate resistance and environmental impacts. Challenges such as insecticide resistance necessitate the exploration of alternative, ecofriendly pesticides. Plant-derived compounds offer sustainable alternatives, with studies demonstrating their efficacy as insecticides and repellents [60]. Insect growth regulators (IGRs) present another avenue for targeted control, inhibiting mosquito development without adverse effects on nontarget organisms [61]. Furthermore, the integration of pheromones in attract-and-kill approaches shows promise in disrupting mosquito life cycles and reducing adult populations [62].

### 4.3. Biological Control Strategies

Biological control methods offer sustainable alternatives to chemical interventions. *Bacillus thuringiensis* and *Bacillus sphaericus* have emerged as environmentally friendly options for larvae control, minimizing nontarget effects [63]. Genetic approaches, including paratransgenesis and vector species modification, show promise in suppressing mosquito populations. For instance, the introduction of *Wolbachia*-infected mosquitoes has demonstrated reduced dengue transmission, highlighting the potential of symbiotic microorganisms as biological control agents [64, 65]. Additionally, the use of predator species such as *Toxorhynchites* and *Copepoda*, generally *Mesocyclops* and *Macrocyclops* species, has shown efficacy in reducing larval populations, though with considerations for environmental risks [66]. RNA interference (RNAi) technology shows potential for targeted mosquito control, offering specificity and effectiveness in laboratory and simulated field trials [61, 67].

## 5. Dengue Surveillance and Reporting Systems Worldwide

The literature highlights the underreporting of dengue cases worldwide and emphasizes the necessity of improved data gathering techniques for surveillance goals [68]. This intricacy results from the fact that the virus has different serotypes and that many data points are needed for thorough surveillance [36]. A minimal set of indicators, such as the quantity of suspected cases, severe cases, fatalities, laboratory-confirmed cases, and circulating serotypes, are advised by the WHO for dengue surveillance [68, 69]. During epidemics, a swift response relies on including suspected patients in monitoring programs promptly, even if laboratory confirmation of cases may take additional time.

A surge of dengue fever since early 2023 has pushed the world close to a record number of cases. Over 5 million infections and 5000 deaths have been reported across 80 countries and territories in five WHO regions (Table 2). The Americas have borne the brunt of this outbreak, accounting for nearly 80% of global cases at over 4.1 million [45]. Europe, once associated only with imported cases, is now witnessing autochthonous transmission, particularly in countries like France and Italy [82]. It is important to emphasize that mandatory reporting in endemic countries is one of the fundamental strategies for dengue surveillance [83]. Countries in the Americas, Europe, and Asia have already adopted mandatory dengue case reporting systems, which have proven to be effective tools for strengthening surveillance and ensuring timely public health responses [84, 85].

In Brazil, for instance, the Notifiable Diseases Information System (SINAN), established in 1990, plays a central role in nationwide dengue surveillance. It supports epidemiological monitoring and decision-making at all levels of government [71, 86]. Compulsory notification is legally required by Law No. 6,259/1975, Decree No. 78,231/1976, and Consolidation Ordinance No. 5/2017, reinforcing the obligation of healthcare professionals to report suspected or confirmed cases [87, 88]. However, the WHO points out that not all countries in the world have laws making this mandatory, such as Japan [89].

Dengue's impact might be surprisingly similar in Africa and the Americas, despite the Americas receiving more historical focus [90]. While Africa has successfully integrated mosquito surveillance into vector control efforts, collaborative efforts like the WAASuN are crucial for improving understanding of mosquito biology, strengthening surveillance, and advocating for increased resources in Africa to combat this widespread disease [70, 91].

In the Eastern Mediterranean Region (EMRO), Pakistan bears a significant burden, having faced recurrent outbreaks since 2006 that affect major cities and result in thousands of cases annually [92]. However, recent studies emphasize that the issue extends beyond Pakistan alone. In particular, the Hindu Kush Himalayan (HKH) region—which includes parts of Afghanistan, Pakistan, India, Nepal, Bhutan, and Bangladesh—has emerged as an important area of concern due to altitudinal shifts in *Aedes*-borne diseases driven by climate change. A systematic review by Sukupayo et al. [93] highlights how changing temperature and precipitation patterns in the HKH are facilitating the expansion of *Aedes aegypti* and *Aedes albopictus* to higher elevations, posing new surveillance and control challenges in previously unaffected areas.

Europe's dengue risk is rising due to the spread of *Aedes albopictus*, a competent vector already established in southern and central regions [94]. These developments have led to intensified prevention efforts, particularly since the 2006 Chikungunya outbreaks [95]. In South Asia, high population density, climate conditions, and infrastructure challenges create a favorable environment for mosquito proliferation. Thailand demonstrates how technology can bolster control strategies—its TanRabad platform has been widely adopted in health policy and outbreak response planning [79, 96].

Dengue cases caused major health concerns in the Western Pacific between 2013 and 2019, with fluctuations ranging from over 430,000 to over 1 million cases annually [97]. Deaths also varied significantly. To predict outbreaks, countries use mosquito monitoring (vector surveillance) but are exploring new models that consider both mosquito activity and human infections for better risk assessment [98].

## 6. Factors Contributing to Dengue Underreporting and Its Effects

Laws and regulations govern notifiable infectious diseases, with the International Health Regulations (IHR) approved by the WHO in 2005 providing guidance for the organization of the international surveillance system [99]. These laws and regulations require healthcare professionals to report the occurrence of these diseases to health surveillance services [100]. However, notification reports are incomplete due to various factors, including diagnostic difficulties and technical failures in the notification registration process, among others, which hinder the recording of information and epidemiological statistics (Table 3) [106].

Notification is a cornerstone of effective health surveillance. In Brazil and other countries, failure to report suspected or confirmed cases can disrupt local-level control actions and delay epidemic responses [68]. Passive surveillance, which is predominant in low- and middle-income settings, is particularly vulnerable to delays in data entry and system access limitations [107, 108].

While predictive models using expansion factors (EFs) offer a method to estimate underreporting, they are often imprecise due to data inequities between active and passive surveillance [30, 68]. Additionally, notification failures can arise from factors such as lack of standardized protocols, incomplete reporting fields, and the absence of national data integration mechanisms [103, 105].

Healthcare workforce–related issues—including insufficient training, lack of familiarity with protocols, and the absence of routine continuing education—remain persistent. Professionals unaware of the relevance of epidemiological data often neglect reporting, especially in overstretched systems [102, 109]. Moreover, a poorly structured notification system may perpetuate inefficient health governance, particularly in resource-limited settings. Political turnover, patronage-based staffing, and inadequate leadership can contribute to institutional weakening and the reemergence of vector-borne diseases [110].

Countries with limited infrastructure and human resources benefit significantly from a robust, agile, and standardized notification system, enabling proactive responses and minimizing economic and social disruptions [111]. International examples, such as Argentina's Laboratory Surveillance System, show that early implementation of structured notification protocols enables timely detection and containment—as seen during the 1997–2000 dengue outbreaks [112]. Conversely, inconsistent data due to underreporting severely hinders the effectiveness of predictive modeling and health planning [113].

## 7. Impact of Underreporting on Public Health

Underreporting dengue has significant consequences for public health systems, especially in low- and middle-income countries (LMICs), where surveillance capacity and healthcare infrastructure are often limited. It is estimated that around 40% of the world's population is at risk of contracting dengue [114, 115]. In countries across Latin America, such as Brazil and Colombia, dengue is a notifiable disease of public interest, and yet studies indicate persistent underreporting, even in the presence of surveillance frameworks [116].

In a globalized world, where the dengue vector closely follows human movement, outbreaks have re-emerged even in countries that had previously eliminated the disease [117]. One notable example is Queensland, Australia, where a 27-day delay in recognizing an imported dengue case led to one of the region's largest outbreaks, illustrating how delayed notification can compromise outbreak response and resource allocation [118].

An effective notification system is therefore essential for outbreak detection, disease containment, and public policy planning [44]. However, underreporting remains prevalent due to diagnostic uncertainty, limited awareness among health professionals, and weaknesses in notification mechanisms [19, 119]. These challenges are further compounded in resource-constrained settings, where local outbreaks may go undetected or be reported late, compromising control measures [120].

To illustrate the diverse impacts of underreporting in different regions, Table 4 summarizes representative examples across countries. The table outlines the nature of underreporting issues, their consequences, and references that support each case.

## 8. Concluding Remarks and Future Perspectives

The influence of climate change, which is related to the increase in global temperature and the oscillation between rainfall periods, is likely to have a greater impact on the epidemiology of dengue when compared to strategies for dealing with the disease [125]. In view of this, the trend is for the American continent to continue to be a region with high rates of dengue, which requires structured planning of the best strategies for its control [108]. According to Marques et al. [120], despite the difficulty in the compulsory notification process, Brazil accounted for nearly 70% of all reported dengue cases in the Americas over the last 5 years [108]. These notifications transform information aimed at better evaluating and monitoring public health actions in Brazil [126]. Despite increased control and compulsory notification, dengue and its complications, such as DHF, are still reemerging as a serious public health problem [49].

An efficient notification process, which produces reliable health data and insights like “Big data,” can generate information crucial for implementing preventive measures leading to control and qualified surveillance, providing statistical and epidemiological data that can predict dengue epidemics in advance [127–129]. These controls could provide the surveillance system with elements to monitor circulating serotypes, anticipate dengue epidemic outbreaks, and thereby reduce the severity of viral syndromes [130, 131]. Indian researchers have analyzed a tool capable of notifying cases of DENV infection in real time to different stakeholders, such as epidemiological surveillance institutions and other health authorities [132]. Systems like this can support the adoption of timely measures to contain dengue cases and can even have a positive impact on the number of notifications.

It is also important to highlight the role of early surveillance systems in breaking the cycle of annual outbreaks. A critical issue discussed by Ghimire [133] is the overwintering of *Aedes* eggs and larvae. These dormant stages can survive in resilient environments and become the source of the first outbreaks in the following year if not properly managed. The lack of adequate surveillance in 1 year not only results in underreported cases but may directly lead to higher transmission rates in the next dengue season. Therefore, surveillance must include the monitoring and elimination of immature vector stages during interepidemic periods, especially in the prewinter and postmonsoon seasons, to avoid resurgence linked to the environmental persistence of vectors. In this context, the study by Sukupayo et al. [134] conducted in central Nepal provides key ecological insights. The authors observed that mosquito abundance and species diversity vary significantly by region, altitude, and season, with *Aedes albopictus* being one of the most prevalent species. Their findings indicate that used tires and shaded containers were major breeding grounds, particularly during the monsoon and postmonsoon seasons. These data reinforce the need for preventive actions focused on environmental management and early surveillance to anticipate dengue outbreaks in high-risk regions.

Arenas et al. [135] emphasize the information from the PAHO regarding dengue in Brazil between 2009 and 2010; it accounted for approximately 60% of all reported dengue cases in the Americas, totaling more than 1 million cases, 1,004,392. The epidemiological bulletin from the Ministry of Health reports a significant increase in dengue cases in São Paulo, showing an approximate 2000% rise in cases when compared to the same period in previous years [135]. These data contribute to the lack of notification and anticipation of health actions, indicating that the efforts exerted by the government to control and contain the disease have not prevented its growth in most states during those years [136]. The difficulty in vector control remains the main cause of this resurgence of the disease [135]. The high number of notifications does not necessarily indicate efficiency in dengue management; we are still inefficient in terms of notifications [137]. As notifications become more effective, coupled with effective public policies in combating dengue, we will achieve greater efficiency in control, helping and relieving the Brazilian population [138].

Effectively addressing the challenge of dengue underreporting requires a multifaceted strategy. This includes enhancing surveillance systems, improving training for healthcare professionals, fostering community involvement, and strengthening public health policies. As we look to the future, it is crucial to align these approaches with significant investments in research and innovation. Advances in artificial intelligence and information technology offer promising solutions to the underreporting of infectious diseases like dengue [116, 139]. These technologies have already revolutionized the field of medicine and have the potential to similarly transform public health monitoring and response [118, 140]. By integrating these advancements, we can significantly improve the detection, reporting, and management of dengue, making this a vital prospect for future efforts.

## 9. Conclusion

This way, we can understand that the underreporting of dengue is a significant challenge that affects not only the statistical approach to health but also the effectiveness of prevention and control measures in disease planning. This study sought to explore the reasons behind underreporting, which may be hindering the disease notification process in various countries worldwide, leading to voluntary underreporting and limiting surveillance systems. We identified the importance of educating the public about dengue and promoting broader awareness of symptoms and prevention measures. However, without a surveillance system that can effectively notify and translate this data into public policies, combating the disease becomes difficult. Within this perspective, we emphasize the need to improve diagnostic capacity, data collection, and reporting by healthcare professionals.

To effectively combat dengue underreporting, it is essential that governments, health organizations, and communities act in a coordinated and collaborative manner. This includes strengthening healthcare infrastructure, promoting research to identify barriers to reporting, and implementing strategies to raise public awareness. Only through sustained joint efforts will it be possible to reduce underreporting and enhance our ability to respond to this pressing public health issue.

Underreporting is often perpetuated by a combination of silent ecological dynamics and systemic limitations. The lack of continuous entomological surveillance enables mosquito populations—particularly dormant *Aedes* eggs and larvae—to persist undetected during the off-season. These hidden reservoirs frequently contribute to early outbreaks in the subsequent year, especially in areas without proactive monitoring and timely vector control interventions.

In this context, dengue underreporting represents a critical obstacle to effective surveillance, which plays a central role in the early detection of outbreaks and epidemics. As we continue to confront global public health challenges, it is essential to recognize that prevention, accurate diagnosis, and timely reporting are key pillars for protecting population health. Tackling dengue underreporting goes beyond statistical accuracy; it is a matter of public health that demands attention, investment, and immediate action.

Although complex, dengue underreporting is not an insurmountable problem. With collective commitment and cross-sector collaboration, it is possible to strengthen our surveillance capacity, improve outbreak preparedness, and reduce the spread of dengue. The health and well-being of populations in Brazil and around the world depend on such integrated efforts.

## Figures and Tables

**Table 1:** Summary of key epidemiological findings on dengue in different regions, including the global landscape, the situation in Latin America, and specifically in Brazil.

**Region**	**Epidemiological findings**	**References**
Global	Dengue has a long history, with records dating back to ancient China.	[40]
	The 20th century saw significant advancements in understanding dengue's etiology and transmission dynamics.	[39, 41, 42]
	Annual infections reach up to 400 million, with an estimated 40,000 deaths attributed to severe dengue.	[43]
	Half of the world's population, approximately 4 billion individuals, reside in areas at risk of dengue transmission.	[44]
	The latest trends highlight the escalating global burden of dengue, with reported cases surging from 500,000 in 2000 to 5.2 million in 2019, spanning 129 countries.	[45]

Latin America	Hyperendemicity and cyclic epidemics occur every 3–5 years.	[39]
	In 2010, over 1.6 million cases of dengue were reported in the Americas.	[46]
	The region documented over 3.1 million cases in 2019, with ongoing increases in reported cases.	[47]
	Subsequent years have witnessed a continuous rise in reported cases, with 2022 recording a staggering 2.8 million cases, representing a significant increase compared to previous years.	[48]
	Nicaragua, Brazil, and Belize reported the highest cumulative incidence rates.	[47]

Brazil	Dengue has a deep-rooted history in Brazil, with the earliest documented case dating back to 1685.	[40, 49]
	Significant epidemics occurred in the late 20th and early 21st centuries, with outbreaks fueled by various dengue virus serotypes.	[50, 51]
	By 1998, a nationwide pandemic swept through Brazil, surpassing 500,000 reported cases, with the highest burden observed in northeast Brazil.	[40]
	Brazil reported over 1.5 million cases in 2024 alone, with the southeast region consistently reporting the highest number of cases.	[52]
	The economic impact of dengue in Brazil is substantial, with estimates reaching $300 million in 2013, underscoring the socioeconomic ramifications of the disease.	[53]

**Table 2:** Number of dengue cases in 2023 and surveillance methods by country and WHO region.

**Region**	**Country**	**Number of cases in 2023** ^**a**^	**National or regional dengue surveillance system**	**References**
Africa	Burkina Faso	146,878	West African Aedes Surveillance Network (WAASuN)	[70]

Americas	Brazil	2,909,404	Notifiable Diseases Information System (SINAN)	[71]
	Peru	271,279	Ecuador–Peru cooperation for climate-informed dengue surveillance	[72]
	Mexico	235,616	Centro Nacional de Programas Preventivos y Control de Enfermedades (CENAPRECE)	[73]

Eastern Mediterranean	Pakistan	20,072	Dengue Expert Advisory Group (DEAG)	[74]

Europe	Italy	82	National Reference Centre for arboviruses (NRC)	[75]
	France	43	National Reference Center (CNR: Centre national de référence) for arboviruses	[76]
	Spain	3	Red Nacional de Vigilancia Epidemiológica (RENAVE)	[77]

Southeast Asia	Bangladesh	308,167	Directorate General of Health Services (DGHS)	[78]
	Thailand	136,655	TanRabad: Software Suite for Dengue Epidemic Surveillance and Control	[79]

Western Pacific	Philippines	167,355	Philippines Integrated Disease Surveillance and Response System	[80]
	Vietnam	149,557	Department of Diseases Control and Prevention	[81]

^a^WHO, Dengue—Global Situation (2023).

**Table 3:** Overview of the main factors that lead to the underreporting of dengue, associating their respective reasons and main impacts.

**Main factors for dengue underreporting**	**Many reasons**	**Main impacts**	**References**
Diagnostic challenges	- Nonspecific symptoms - Cross-reactivity with other arboviruses - Limited access to RT-PCR and rapid tests	- Low case confirmation - Misdiagnosis and improper treatment	[101–103]
Lack of professional training	- Insufficient knowledge of dengue recognition - Lack of training in reporting procedures	- Missed or late notifications - Inaccurate surveillance data	[68, 102]
Weak notification system	- Inadequate IT infrastructure - Manual notification processes - Low supervision of case reporting	- Fragmented data - Limited ability to monitor trends and respond effectively	[104]
Policy and governance gaps	- Lack of sustained investment - Reduced focus during interepidemic periods	- Weak public health response - Reduced community	[104]
Human resource constraints	- High staff turnover - Newly graduated or untrained staff - Politically motivated hiring	- Loss of institutional knowledge - Collapse of consistent reporting	[103, 105]

Abbreviation: RT-PCR, reverse transcription polymerase chain reaction.

**Table 4:** International examples of dengue underreporting impacts.

**Country/region**	**Underreporting issue**	**Impact**	**References**
Brazil	Approximately 95% underreporting during epidemics; large discrepancy in dengue hemorrhagic fever cases	Public costs increased from BRL 164 M to BRL 447 M; total social loss estimated at $1.2B	[111, 121]
Colombia	Notification data suggest systematic underreporting of suspected cases	Hinders identification of true disease burden and planning of public policies	[122]
Bangladesh	Reduced severity of reported cases; underreporting mitigated by serological confirmation	Improved control via awareness and testing; highlights importance of accurate reporting	[123]
Australia (Queensland)	27-day delay in recognizing imported case; resulted in major local outbreak	Significant local outbreak; delayed response caused health and financial burdens	[124]
Global	Official WHO estimates of 100 M infections/year vs. modeling estimates of 390 M	Global planning based on inaccurate figures; distorts allocation of resources	[44]

## Data Availability

Data sharing is not applicable to this article as no new data were created or analyzed in this study.

## References

[1] Weaver S. C. (2013). Urbanization and Geographic Expansion of Zoonotic Arboviral Diseases: Mechanisms and Potential Strategies for Prevention. *Trends in Microbiology*.

[2] Okoye E. C., Mitra A. K., Lomax T., Nunaley C. (2024). Dengue Fever Epidemics and the Prospect of Vaccines: A Systematic Review and Meta-Analysis Using Clinical Trials in Children. *Diseases*.

[3] Gloria-Soria A., Ayala D., Bheecarry A. (2016). Global Genetic Diversity of *Aedes aegypti*. *Molecular Ecology*.

[4] Abuelmaali S. A., Mashlawi A. M., Ishak I. H. (2024). Population Genetic Structure of Aedes aegypti Subspecies in Selected Geographical Locations in Sudan. *Scientific Reports*.

[5] Abuelmaali S. A., Jamaluddin J. A. F., Noaman K. (2021). Distribution and Genetic Diversity of Aedes aegypti Subspecies Across the Sahelian Belt in Sudan. *Pathogens*.

[6] Burkett-Cadena N. D., Vittor A. Y. (2018). Deforestation and Vector-Borne Disease: Forest Conversion Favors Important Mosquito Vectors of Human Pathogens. *Basic and Applied Ecology*.

[7] Ortiz D. I., Piche-Ovares M., Romero-Vega L. M., Wagman J., Troyo A. (2022). The Impact of Deforestation, Urbanization, and Changing Land Use Patterns on the Ecology of Mosquito and Tick-Borne Diseases in Central America. *Insects*.

[8] Madewell Z. J. (2020). Arboviruses and Their Vectors. *Southern Medical Journal*.

[9] Petryna A. (2015). *International Encyclopedia of the Social & Behavioral Sciences*.

[10] Institute of Medicine (US) Committee for the Study of the Future of Public Health (1988). *The future of Public Health*.

[11] Riley L. W., Blanton R. E. (2018). Advances in Molecular Epidemiology of Infectious Diseases: Definitions, Approaches, and Scope of the Field. *Microbiology Spectrum*.

[12] Holveck J. C., Ehrenberg J. P., Ault S. K. (2007). Prevention, Control, and Elimination of Neglected Diseases in the Americas: Pathways to Integrated, Inter-Programmatic, Inter-Sectoral Action for Health and Development. *BMC Public Health*.

[13] WHO Intervention Strategies. https://www.who.int/teams/control-of-neglected-tropical-diseases/interventions/strategies.

[14] Mendis K. (2019). Mass Drug Administration Should Be Implemented as a Tool to Accelerate Elimination: Against. *Malaria Journal*.

[15] Barata R. B. (2022). Vigilância epidemiológica: breve histórico e A experiência dos Estados Unidos e do estado de São Paulo. *Epidemiol Serv Saude*.

[16] Chow A., Leo Y.-S. (2017). *International Encyclopedia of Public Health*.

[17] Baker M. G., Easther S., Wilson N. (2010). A Surveillance Sector Review Applied to Infectious Diseases at a Country Level. *BMC Public Health*.

[18] Guzman M. G., Gubler D. J., Izquierdo A., Martinez E., Halstead S. B. (2016). Dengue Infection. *Nature Reviews Disease Primers*.

[19] Isere E., Fatiregun A., Ajayi I. (2015). An Overview of Disease Surveillance and Notification System in Nigeria and the Roles of Clinicians in Disease Outbreak Prevention and Control. *Nigerian Medical Journal*.

[20] Jamison D. T., Breman J. G., Measham A. R. (2006). Disease Control Priorities in Developing Countries.

[21] Gilbert R., Cliffe S. J. (2016). *Public Health Intelligence*.

[22] Silva M. M. O., Rodrigues M. S., Paploski I. A. D. (2016). Accuracy of Dengue Reporting by National Surveillance System, Brazil. *Emerging Infectious Diseases*.

[23] Silva D. M., Curcio J. S., Silva L. D. (2024). Detection of Arboviruses in *Aedes aegypti* Through Transovarian Analysis: A Study in Goiânia, Goiás. *Revista da Sociedade Brasileira de Medicina Tropical*.

[24] Diaz-Quijano F. A. (2015). Dengue Severity: A Key Determinant of Underreporting. *Tropical Medicine & International Health*.

[25] Gibbons C. L., Mangen M.-J. J., Plass D. (2014). Measuring Underreporting and Under-Ascertainment in Infectious Disease Datasets: A Comparison of Methods. *BMC Public Health*.

[26] WHO Vector-Borne Diseases. https://www.who.int/news-room/fact-sheets/detail/vector-borne-diseases.

[27] Khan M. B., Yang Z.-S., Lin C.-Y. (2023). Dengue Overview: An Updated Systemic Review. *Journal of Infection and Public Health*.

[28] Soni S., Gill V. J. S., Anusheel (2023). Dengue, Chikungunya, and Zika: The Causes and Threats of Emerging and Re-Emerging Arboviral Diseases. *Cureus*.

[29] Undurraga E. A., Betancourt-Cravioto M., Ramos-Castañeda J. (2015). Economic and Disease Burden of Dengue in Mexico. *PLoS Neglected Tropical Diseases*.

[30] Undurraga E. A., Halasa Y. A., Shepard D. S. (2013). Use of Expansion Factors to Estimate the Burden of Dengue in Southeast Asia: A Systematic Analysis. *PLoS Neglected Tropical Diseases*.

[31] Amarasinghe A. (2011). Dengue Virus Infection in Africa. *Emerging Infectious Diseases*.

[32] Nunez-Avellaneda D., Tangudu C., Barrios-Palacios J. (2021). Co-Circulation of All Four Dengue Viruses and Zika Virus in Guerrero, Mexico, 2019. *Vector-Borne and Zoonotic Diseases*.

[33] Alghsham R. S., Shariq A., Rasheed Z. (2023). Dengue: A Global Health Concern. *International Journal of Health Sciences*.

[34] Kularatne S. A., Dalugama C. (2022). Dengue Infection: Global Importance, Immunopathology and Management. *Clinical Medicine*.

[35] Murugesan A., Manoharan M. (2020). *Emerging and Reemerging Viral Pathogens*.

[36] Sirisena N., Mahilkar S., Sharma C., Jain J., Sunil S. (2021). Concurrent Dengue Infections: Epidemiology & Clinical Implications. *Indian Journal of Medical Research*.

[37] Raza M. A., Khan M. A., Ejaz K., Haider M. A., Rasheed F. (2020). A Case of Dengue Fever With Hemorrhagic Manifestations. *Cureus*.

[38] Raafat N., Blacksell S. D., Maude R. J. (2019). A Review of Dengue Diagnostics and Implications for Surveillance and Control. *Transactions of The Royal Society of Tropical Medicine and Hygiene*.

[39] Murray N. E. A., Quam M. B., Wilder-Smith A. (2013). Epidemiology of Dengue: Past, Present and Future Prospects. *Clinical Epidemiology*.

[40] Salles T. S., da Encarnação Sá-Guimarães T., de Alvarenga E. S. L. (2018). History, Epidemiology and Diagnostics of Dengue in the American and Brazilian Contexts: A Review. *Parasit Vectors*.

[41] Silva N. M., Santos N. C., Martins I. C. (2020). Dengue and Zika Viruses: Epidemiological History, Potential Therapies, and Promising Vaccines. *Tropical Medicine and Infectious Disease*.

[42] Chen R., Vasilakis N. (2011). Dengue — Quo tu et quo vadis?. *Viruses*.

[43] CDC Data and Maps. https://www.cdc.gov/dengue/statistics-maps/data-and-maps.html#:%7E:text=Almost%2520half%2520of%2520the%2520world%2527s,40%252C000%2520die%2520from%2520severe%2520dengue.

[44] WHO Dengue and Severe Dengue. https://www.who.int/health-topics/dengue-and-severe-dengue#tab=tab_1.

[45] WHO Dengue - Global Situation. https://www.who.int/emergencies/disease-outbreak-news/item/2023-DON498.

[46] Dick O. B., San Martín J. L., Montoya R. H., del Diego J., Zambrano B., Dayan G. H. (2012). The History of Dengue Outbreaks in the Americas. *American Journal of Tropical Medicine and Hygiene*.

[47] WHO Geographical Expansion of Cases of Dengue and Chikungunya Beyond the Historical Areas of Transmission in the Region of the Americas. https://www.who.int/emergencies/disease-outbreak-news/item/2023-DON498.

[48] Mohapatra R. K., Bhattacharjee P., Desai D. N. (2024). Global Health Concern on the Rising Dengue and Chikungunya Cases in the American Regions: Countermeasures and Preparedness. *Health Science Reports*.

[49] Fares R. C. G., Souza K. P. R., Añez G., Rios M. (2015). Epidemiological Scenario of Dengue in Brazil. *BioMed Research International*.

[50] Nogueira R. M. R., Miagostovich M. P., Schatzmayr H. G. (1999). Dengue in the State of Rio de Janeiro, Brazil, 1986-1998. *Memórias do Instituto Oswaldo Cruz*.

[51] Romano C. M., de Matos A. M., Araújo E. S. A. (2010). Characterization of Dengue Virus Type 2: New Insights on the 2010 Brazilian Epidemic. *PLoS One*.

[52] ECDC Dengue Worldwide Overview. https://www.ecdc.europa.eu/en/dengue-monthly.

[53] Bavia L., Melanda F. N., de Arruda T. B. (2020). Epidemiological Study on Dengue in Southern Brazil Under the Perspective of Climate and Poverty. *Scientific Reports*.

[54] Wong J., Stoddard S. T., Astete H., Morrison A. C., Scott T. W. (2011). Oviposition Site Selection by the Dengue Vector Aedes aegypti and Its Implications for Dengue Control. *PLoS Neglected Tropical Diseases*.

[55] Amierul M., Mahmud F., Hatta M. (2023). The Application of Environmental Management Methods in Combating Dengue: A Systematic Review. *Environmental Health Research*.

[56] Amierul M., Mahmud F., Hatta M. (2019). Environmental Management for Dengue Control: A Systematic Review Protocol. *BMJ Open*.

[57] James L. D., Winter N., Stewart A. T. M. (2022). Field trials reveal the complexities of deploying and evaluating the impacts of yeast-baited ovitraps on Aedes mosquito densities in Trinidad, West Indies. *Scientific Reports*.

[58] Duncombe J., Hu W., Clements A., Ritchie S., Weinstein P., Espino F. E. (2012). Geographical Information Systems for Dengue Surveillance. *American Journal of Tropical Medicine and Hygiene*.

[59] Goellner E., Neckel A., Bodah B. W. (2021). Geospatial Analysis of Ae. aegypti Foci in Southern Brazil. *Journal of Environmental Chemical Engineering*.

[60] Silvério M. R. S., Espindola L. S., Lopes N. P., Vieira P. C. (2020). Plant Natural Products for the Control of Aedes aegypti: The Main Vector of Important Arboviruses. *Molecules*.

[61] Rather I. A., Parray H. A., Lone J. B. (2017). Prevention and Control Strategies to Counter Dengue Virus Infection. *Frontiers in Cellular and Infection Microbiology*.

[62] Ong S.-Q., Jaal Z. (2015). Investigation of mosquito oviposition pheromone as lethal lure for the control of *Aedes aegypti* (L.)(Diptera: Culicidae). *Parasites & Vectors*.

[63] Jing Q., Wang M. (2019). Dengue Epidemiology. *Global Health Journal*.

[64] Niang E. H. A., Bassene H., Fenollar F., Mediannikov O. (2018). Biological Control of Mosquito-Borne Diseases: The Potential of *Wolbachia* -Based Interventions in an IVM Framework. *Journal of Tropical Medicine*.

[65] Zohra T., Khalil A. T., Saeed F. (2022). Green Nano-Biotechnology: A New Sustainable Paradigm to Control Dengue Infection. *Bioinorganic Chemistry and Applications*.

[66] Huang Y.-J., Higgs S., Vanlandingham D. (2017). Biological Control Strategies for Mosquito Vectors of Arboviruses. *Insects*.

[67] Yadav M., Dahiya N., Sehrawat N. (2023). Mosquito Gene Targeted RNAi Studies for Vector Control. *Functional & Integrative Genomics*.

[68] Angelo M., Ramalho W. M., Gurgel H., Belle N., Pilot E. (2020). Dengue Surveillance System in Brazil: A Qualitative Study in the Federal District. *International Journal of Environmental Research and Public Health*.

[69] WHO Monitoring and Evaluation of Programmes. https://www.who.int/teams/control-of-neglected-tropical-diseases/dengue-and-severe-dengue/monitoring-and-evaluation-of-programmes.

[70] Dadzie S. K., Akorli J., Coulibaly M. B. (2022). Building the Capacity of West African Countries in Aedes Surveillance: Inaugural Meeting of the West African Aedes Surveillance Network (WAASuN). *Parasit Vectors*.

[71] Rocha M. S., Bartholomay P., Cavalcante M. V. (2020). Sistema de Informação de Agravos de Notificação (Sinan): principais características da notificação e da análise de dados relacionada à tuberculose. *Epidemiologia e Serviços de Saúde*.

[72] Viennet E., Harley D. (2017). Climate Services for Health: Cooperation for Climate Informed Dengue Surveillance. *Lancet Planetary Health*.

[73] Sanchez Tejeda G., Benitez Valladares D., Correa Morales F. (2023). Early Warning and Response System for Dengue Outbreaks: Moving From Research to Operational Implementation in Mexico. *PLOS Global Public Health*.

[74] Dengue Expert Advisory Group (DEAG) Dengue GCP Guidelines. https://deag.punjab.gov.pk/system/files/Dengue%2520GCP%2520GUIDELINES%25202020.pdf.

[75] Vita S., Lalle E., Caputi P. (2024). Dengue Fever as Autochthonous Infectious Disease in Italy: Epidemiological, Clinical and Virological Characteristics. *Travel Medicine and Infectious Disease*.

[76] Cochet A., Calba C., Jourdain F. (2022). Autochthonous Dengue in Mainland France, 2022: Geographical Extension and Incidence Increase. *Eurosurveillance*.

[77] Saravia Campelli G., García Expósito E., Antonio del Castillo J. (2023). Agrupación de casos de dengue autóctono en Ibiza. https://www.researchgate.net/publication/370156667_Agrupacion_de_casos_de_dengue_autoctono_en_Ibiza.

[78] Amin M. R., Islam M. R., Bhuiyan M. (2022). Sketch of 2018 Dengue Outbreak in a Megacity, Bangladesh. *Tropical Medicine and Health*.

[79] Sahavechaphan N., Rattananen M., Panichphol P., Wongwilai W., Iamsiri S., Sadakorn P. (2016). TanRabad: Software Suite for Dengue Epidemic Surveillance and Control. *International Journal of Infectious Diseases*.

[80] Undurraga E. A., Edillo F. E., Erasmo J. N. V. (2017). Disease Burden of Dengue in the Philippines: Adjusting for Underreporting by Comparing Active and Passive Dengue Surveillance in Punta Princesa, Cebu City. *American Journal of Tropical Medicine and Hygiene*.

[81] Taurel A.-F., Luong C. Q., Nguyen T. T. T. (2023). Age Distribution of Dengue Cases in Southern Vietnam From 2000 to 2015. *PLoS Neglected Tropical Diseases*.

[82] ECDC Autochthonous Vectorial Transmission of Dengue Virus in Mainland EU/EEA, 2010-Presen. https://www.ecdc.europa.eu/en/all-topics-z/dengue/surveillance-and-disease-data/autochthonous-transmission-dengue-virus-eueea.

[83] Beatty M. E., Stone A., Fitzsimons D. W. (2010). Best Practices in Dengue Surveillance: A Report From the Asia-Pacific and Americas Dengue Prevention Boards. *PLoS Neglected Tropical Diseases*.

[84] PAHO Dengue Indicators. https://opendata.paho.org/en/dengue-indicators.

[85] ECDC Dengue Annual Epidemiological Report for 2021. Stockholm 2023. https://www.ecdc.europa.eu/en/publications-data/dengue-annual-epidemiological-report-2022.

[86] Assis V. C., Amaral M. D. P. H. D., Mendonça A. E. D. (2014). *Análise da qualidade das notificações de dengue informadas no sistema de informação de agravos de notificação, na epidemia de 2010, em uma cidade polo da Zona da Mata de estado de Minas Gerais*.

[87] Lima C. R., Schramm J. M., Coeli C. M., Silva M. E. M. (2009). Revisão das dimensões de qualidade dos dados e métodos aplicados na avaliação dos sistemas de informação em saúde. *Cadernos de Saúde Pública*.

[88] World Health Organization Framework and Standards for Country Health Information Systems. https://www.who.int/publications-detail-redirect/9789241595940.

[89] WHO Dengue - Global Situation. https://www.who.int/emergencies/disease-outbreak-news/item/2023-DON498.

[90] Gainor E. M., Harris E., LaBeaud A. D. (2022). Uncovering the Burden of Dengue in Africa: Considerations on Magnitude, Misdiagnosis, and Ancestry. *Viruses*.

[91] Coulibaly Z. I., Gowelo S., Traore I. (2023). Strengthening Adult Mosquito Surveillance in Africa for Disease Control: Learning From the Present. *Current Opinion in Insect Science*.

[92] Ghouth S. B. (2018). Dengue in the WHO Eastern Mediterranean Region: Challenges to Understand Its Epidemiology. *Health and Primary Care*.

[93] Sukupayo P. R., Poudel R. C., Ghimire T. R. (2024). Nature’s Solution to *Aedes* Vectors: *Toxorhynchites* as a Biocontrol Agent. *Journal of Tropical Medicine*.

[94] Brem J., Elankeswaran B., Erne D. (2024). Dengue “Homegrown” in Europe (2022 to 2023). *New Microbes and New Infections*.

[95] Schaffner F., Medlock J. M., Bortel W. (2013). Public Health Significance of Invasive Mosquitoes in Europe. *Clinical Microbiology and Infection*.

[96] Knerer G., Currie C. S. M., Brailsford S. C. (2021). Reducing Dengue Fever Cases at the Lowest Budget: A Constrained Optimization Approach Applied to Thailand. *BMC Public Health*.

[97] Togami E., Chiew M., Lowbridge C. (2023). Epidemiology of Dengue Reported in the World Health Organization Western Pacific Region, 2013–2019. *Western Pacific Surveillance and Response Journal*.

[98] Chang M. S., Christophel E. M., Gopinath D. (2011). Challenges and Future Perspective for Dengue Vector Control in the Western Pacific Region. *Western Pacific Surveillance and Response*.

[99] Fidler D. P. (2005). From International Sanitary Conventions to Global Health Security: The New International Health Regulations. *Chinese Journal of International Law*.

[100] CDC International Health Regulations. https://www.cdc.gov/nndss/about/ihr.html.

[101] Caicedo-Borrero D. M., Tovar J. R., Méndez A. (2020). Development and Performance of Dengue Diagnostic Clinical Algorithms in Colombia. *American Journal of Tropical Medicine and Hygiene*.

[102] Tsheten T., Clements A. C. A., Gray D. J., Gyeltshen K., Wangdi K. (2021). Medical Practitioner’s Knowledge on Dengue Management and Clinical Practices in Bhutan. *PLoS One*.

[103] Kolawole O., Seriki A., Irekeola A., Ogah J. (2018). The Neglect and Fast Spread of Some Arboviruses: A Note for Healthcare Providers in Nigeria. *Diseases*.

[104] Barbalho I. M. P., Fernandes F., Barros D. M. S. (2022). Electronic Health Records in Brazil: Prospects and Technological Challenges. *Public Health*.

[105] Sarti E., L’Azou M., Mercado M. (2016). A Comparative Study on Active and Passive Epidemiological Surveillance for Dengue in Five Countries of Latin America. *International Journal of Infectious Diseases*.

[106] Amaku M., Burattini M. N., Chaib E. (2017). Estimating the Prevalence of Infectious Diseases From Under-Reported Age-Dependent Compulsorily Notification Databases. *Theoretical Biology and Medical Modelling*.

[107] Da Silva G. A., De Oliveira C. M. G. (2014). O registro das doenças de notificação compulsória: A participação dos profissionais da saúde e da comunidade. *Revista de Epidemiologia e Controle de Infecção*.

[108] Lenharo M. (2023). Dengue Is Breaking Records in the Americas — What’s Behind the Surge?. *Nature*.

[109] Mohammed Yusuf A., Abdurashid Ibrahim N. (2019). Knowledge, Attitude and Practice Towards Dengue Fever Prevention and Associated Factors Among Public Health Sector Health-Care Professionals: In Dire Dawa, Eastern Ethiopia. *Risk Management and Healthcare Policy*.

[110] Guimarães L. M., Cunha G. M. (2020). Diferenças por sexo e idade no preenchimento da escolaridade em fichas de vigilância em capitais brasileiras com maior incidência de dengue, 2008-2017. *Cadernos de Saúde Pública*.

[111] Martelli C. M. T., Siqueira J. B., Parente M. P. P. D. (2015). Economic Impact of Dengue: Multicenter Study Across Four Brazilian Regions. *PLoS Neglected Tropical Diseases*.

[112] Morgan O. W., Pinner R. W. (2009). *Encyclopedia of Microbiology*.

[113] Carabali M., Jaramillo-Ramirez G. I., Rivera V. A., Mina Possu N.-J., Restrepo B. N., Zinszer K. (2021). Assessing the Reporting of Dengue, Chikungunya and Zika to the National Surveillance System in Colombia From 2014–2017: A Capture-Recapture Analysis Accounting for Misclassification of Arboviral Diagnostics. *PLoS Neglected Tropical Diseases*.

[114] Cobo F. (2014). *Imported Infectious Diseases*.

[115] Lessa C. L. S., Hodel K. V. S., Gonçalves M. . S., Machado B. A. S. (2023). Dengue as a Disease Threatening Global Health: A Narrative Review Focusing on Latin America and Brazil. *Tropical Medicine and Infectious Disease*.

[116] Herbuela V. R. D. M., Karita T., Francisco M. E., Watanabe K. (2020). An Integrated mHealth App for Dengue Reporting and Mapping, Health Communication, and Behavior Modification: Development and Assessment of Mozzify. *JMIR Formative Research*.

[117] Shaw W. R., Catteruccia F. (2019). Vector Biology Meets Disease Control: Using Basic Research to Fight Vector-Borne Diseases. *Nature Microbiology*.

[118] Beam A. L., Drazen J. M., Kohane I. S., Leong T.-Y., Manrai A. K., Rubin E. J. (2023). Artificial Intelligence in Medicine. *New England Journal of Medicine*.

[119] Murray J., Cohen A. L. (2017). *International Encyclopedia of Public Health*.

[120] Marques C. A., Portugal F. B. (2020). Assessment of the Lack of Completeness of Compulsory Dengue Fever Notifications Registered by a Small Municipality in Brazil. *Cien Saude Colet*.

[121] Junior J. B. S., Massad E., Lobao-Neto A., Kastner R., Oliver L., Gallagher E. (2022). Epidemiology and Costs of Dengue in Brazil: A Systematic Literature Review. *International Journal of Infectious Diseases*.

[122] Moreno Gomez G. A., Moreno Gómez J. G., Cabezas Restrepo Á. M., MúneraBenavides J. E., Ocampo Alzate K. P., Moreno Villegas V. (2016). Cumplimiento en la notificación de casos probables de dengue en el Área Metropolitana Centro Occidente de Colombia en el año 2014. *Revista Médica de Risaralda*.

[123] Sharmin S., Viennet E., Glass K., Harley D. (2015). The Emergence of Dengue in Bangladesh: Epidemiology, Challenges and Future Disease Risk. *Transactions of The Royal Society of Tropical Medicine and Hygiene*.

[124] Ritchie S. A., Pyke A. T., Hall-Mendelin S. (2013). An explosive epidemic of DENV-3 in Cairns, Australia. *PLoS One*.

[125] Barcellos C., Matos V., Lana R. M., Lowe R. (2024). Climate Change, Thermal Anomalies, and the Recent Progression of Dengue in Brazil. *Scientific Reports*.

[126] https://bvsms.saude.gov.br/bvs/publicacoes/health_brazil_2015_2016.pdf.

[127] Corsi A., de Souza F. F., Pagani R. N., Kovaleski J. L. (2021). Big Data Analytics as a Tool for Fighting Pandemics: A Systematic Review of Literature. *Journal of Ambient Intelligence and Humanized Computing*.

[128] Jiao Z., Ji H., Yan J., Qi X. (2023). Application of Big Data and Artificial Intelligence in Epidemic Surveillance and Containment. *Intelligent Medicine*.

[129] Razzak M. I., Imran M., Xu G. (2020). Big Data Analytics for Preventive Medicine. *Neural Computing and Applications*.

[130] Gubler D. J. (1998). Dengue and Dengue Hemorrhagic Fever. *Clinical Microbiology Reviews*.

[131] Dolley S. (2018). Big Data’s Role in Precision Public Health. *Frontiers in Public Health*.

[132] Sood S. K., Sood V., Mahajan I., Sahil (2023). an Intelligent Healthcare System for Predicting and Preventing Dengue Virus Infection. *Computing*.

[133] Ghimire T. R. (2023). Dengue Skyrocket: Should It Be Neglected Now?. *International Journal of Medical Parasitology and Epidemiology Sciences*.

[134] Sukupayo P. R., Poudel R. C., Ghimire T. R. (2025). Entomological Surveillance of Container-Breeding Mosquitoes Focusing on *Aedes* (*Stegomyia*) (Diptera: Culicidae) Vectors Along Altitudinal Range in Nepal. *Journal of Medical Entomology*.

[135] Villabona Arenas C. J., Botelho A. V., Botelho A. C., Passos S. D. (2012). The Burden of Dengue: Jundiaí, Brazil–January 2010. *Revista da Associação Médica Brasileira (English Edition)*.

[136] Nnebue C., Onwasigwe C., Onyeonoro U., Adogu P. U. (2012). Awareness and Knowledge of Disease Surveillance and Notification by Health-Care Workers and Availability of Facility Records in Anambra state, Nigeria. *Nigerian Medical Journal*.

[137] Ladner J., Rodrigues M., Davis B., Besson M.-H., Audureau E., Saba J. (2017). Societal Impact of Dengue Outbreaks: Stakeholder Perceptions and Related Implications. A Qualitative Study in Brazil, 2015. *PLoS Neglected Tropical Diseases*.

[138] Melo G. B. T., Angulo-Tuesta A., da S.,. E. N., Obara M. T. (2023). Funding for Research on Dengue in Brazil, 2004-2020. *Saúde Em Debate*.

[139] Paganelli A. I., Mondéjar A. G., da Silva A. C. (2022). Real-Time Data Analysis in Health Monitoring Systems: A Comprehensive Systematic Literature Review. *Journal of Biomedical Informatics*.

[140] Alowais S. A., Alghamdi S. S., Alsuhebany N. (2023). Revolutionizing Healthcare: The Role of Artificial Intelligence in Clinical Practice. *BMC Medical Education*.

